# Postoperative Chemoradiotherapy Combined with Epirubicin-Based Triplet Chemotherapy for Locally Advanced Adenocarcinoma of the Stomach or Gastroesophageal Junction

**DOI:** 10.1371/journal.pone.0054233

**Published:** 2013-01-25

**Authors:** Guichao Li, Zhen Zhang, Xuejun Ma, Ji Zhu, Gang Cai

**Affiliations:** Departments of Radiation Oncology and Oncology, Shanghai Cancer Center, Shanghai Medical College, Fudan University, Shanghai, China; Faculté de médecine de Nantes, France

## Abstract

**Background:**

Due to low tolerance to chemotherapy, the maximum number of cycles of postoperative adjuvant chemotherapy is 4 in adjuvant gastric clinical trials. The aim of this study is to retrospectively evaluate the safety and efficacy of adjuvant epirubicin-based triplet chemotherapy and radiotherapy in the treatment of resected locally advanced stomach or gastroesophageal junction adenocarcinoma.

**Methodology/Principal Findings:**

From January 2004 to July 2008, ninety-seven consecutive gastric or gastroesophageal junction adenocarcinoma patients in stages T3–4/N+ were treated with postoperative radiotherapy and chemotherapy. The recommended treatment plan was radical resection followed by 1–2 cycles of adjuvant chemotherapy (ACT), postoperative chemoradiotherapy (CRT), and, finally, 4–5 cycles of ACT. The patients were classified into two groups depending on the number of cycles of ACT: group 1 received 4–6 cycles (n = 59), and group 2 received 0–3 cycles (n = 38). The detailed grouping is as follows: RT alone, 2; RT and CT, 18; concurrent RTCT and CT, 41; and CRT, 36. Of the 97 patients, 77 patients received concurrent therapy (CRT, (5-fluorouracil or capecitabine), and 20 received radiotherapy alone because of patient refusal (n = 15) or treatment toxicity (n = 5). After a median follow-up of 44 months, the 3-year disease free survival(DFS) and overall survival (OS) were 66.5% and 69.5% for group 1 and 45.5% and 50% for group 2, respectively (p = 0.005 and p = 0.024). Multivariate analysis revealed that 4–6 cycles of ACT, lymphovascular invasion, or peritoneal metastasis were independent prognostic factors for disease-free survival or overall survival (p<0.05).

**Conclusions/Significance:**

This study demonstrates that concurrent chemoradiation with adjuvant epirubicin-based triplet chemotherapy is feasible and tolerable for gastric or gastroesophageal junction carcinoma patients. Patients can benefit from more cycles of ACT.

## Introduction

Gastric cancer is the fourth most frequently diagnosed cancer worldwide and accounts for 8% of all new cancer diagnoses. Gastric cancer is responsible for 10% of all cancer deaths, and it is one of the most commonly diagnosed malignancies in Asia [1], [2]. Most patients with gastric cancer present at an advanced stage, and the prognosis remains poor, particularly in more advanced stages [3], [4].

Surgery is the primary gastric cancer treatment, but surgical treatment alone has a high rate of locoregional and distant recurrence [3], [4], and significant research has focused on identifying effective adjuvant therapies to reduce the risk of relapse after surgery. A meta-analysis of postoperative adjuvant chemotherapy (ACT) showed moderate survival benefits [5]–[9], and five-year follow-up data of an ACTS-GC trial [10] showed that postoperative adjuvant therapy with S-1 can improve overall survival and relapse-free survival in patients with stage II or III gastric cancer who had undergone D2 gastrectomy. In addition, the Gastric Surgical Adjuvant Trial INT 0116 [11] showed that relapse-free-survival (p<0.001) and overall survival (p = 0.005) benefit from adjuvant CRT for patients with a high risk of relapse. In that study, the concurrent chemotherapy regimen was 5-fluorouracil (5-FU) plus leucovorin, but this regimen is now thought to be insufficient for preventing remote metastasis. A regimen of epirubicin, cisplatin, and 5-fluorouracil (ECF) has been increasingly used in advanced disease and has been investigated in the adjuvant setting in phase II studies. The Medical Research Council Adjuvant Gastric Infusional Chemotherapy (MAGIC) study [12] demonstrated that a perioperative regimen of ECF decreased tumor size and stage and significantly improved progression-free survival (PFS) and overall survival (OS) in operable gastric cancer or lower esophageal adenocarcinoma. The subsequent REAL-2 trial confirmed that in the epirubicin-based triple-regimen [epirubicin (50 mg/m^2^ on Day 1)+cisplatin (30 mg/m^2^ on Days 1–3)+5-FU (425 mg/m^2^/day on Days 1–5)], the substitution of cisplatin with oxaliplatin and of 5-FU with capecitabine resulted in lower toxicity [13]. The recently reported Cancer and Leukemia Group B (CALGB) 80101 trial demonstrated a similar outcome with ECF and 5-FU (ASCO 2011). However, the efficacy of CRT with optimal cycles of chemotherapy has not been well studied; this information is necessary to optimize the treatment of locally advanced gastric cancer. The aim of this study is to evaluate the efficacy of adjuvant CRT and the effect of cycle number in adjuvant epirubicin-based chemotherapy in patients with Stage T3–4/N+ gastric or gastroesophageal junction adenocarcinoma.

## Methods and Materials

### 1 Study subjects

All data were collected from consenting individuals according to the protocols approved by the Ethics Review Board at Fudan University Shanghai Cancer Center. From January 2004 to July 2008, 97 consecutive patients diagnosed with locally advanced and non-metastatic adenocarcinoma of the stomach or gastroesophageal junction were enrolled in this study. Pathologic diagnoses were obtained in all cases before staging and treatment. The workup consisted of a complete history, physical examination, performance status, complete blood count, liver and renal function tests, endoscopy, chest, abdominal computed tomography (CT) scan, and ultrasonography of the pelvis. Within the cohort, 59 patients (group 1) were treated according to an institutional clinical protocol that consisted of surgery followed by adjuvant chemo/radiotherapy and 4–6 cycles of adjuvant epirubicin-based chemotherapy. The remaining 38 patients (group 2) were treated with surgery followed by chemo/radiotherapy and 0–3 cycles of ACT using the same regimen because of early disease progression (n = 3), patient refusal (n = 15), or treatment toxicity (n = 20). The patient characteristics are listed in Table 1; there were no significant differences between the two groups.

**Table 1:** Patient characteristics.

Variable	CRT*+ACT†(4–6)		CRT+ACT(0–3)		?^2^	P
	n	%	n	%		
Age (yr)			0.001	0.975		
Median	54		52			
<50	20	(34)	13	(34)		
≥50	39	(66)	25	(66)		
Gender			2.238	0.135		
Male	40	(68)	31	(82)		
Female	19	(32)	7	(18)		
Pathologic type						
Adenocarcinoma	59	(100)	38	(100)		
Tumor location			0.462	0.497		
Cardial	14	(24)	7	(18)		
Body	30	(51)	16	(42)		
Antrum/pylorus	15	(25)	15	(40)		
pT stage			1.260	0.739		
T1	2	(3)	1	(3)		
T2	10	(17)	6	(16)		
T3	37	(63)	21	(55)		
T4	10	(17)	10	(27)		
pN stage			3.848	0.278		
N0	8	(14)	4	(11)		
N1	30	(51)	17	(44)		
N2	20	(34)	13	(34)		
N3	1	(1)	4	(11)		
Positive LN ratio			0.372	0.542		
≥0.3	32	(54)	23	(60)		
<0.3	27	(46)	15	(40)		
Overall stage (AJCC††)			3.074	0.38		
Ib	2	(3)	1	(3)		
II	9	(16)	9	(24)		
III	38	(64)	18	(47)		
IV	10	(17)	10	(26)		
Residual disease			2.029	0.154		
R0§	57	(97)	34	(89)		
R1∥	2	(3)	4	(11)		
Surgery			0.181	0.683		
D2¶	30	(51)	21	(55)		
D1#	29	(49)	17	(45)		
Concurrent CT			0.007	0.932		
With CCT	47	30				
Without CCT	12	8				
ECOG			0.146	0.702		
0	15	(25)	11	(29)		
1	44	(75)	27	(71)		

* : CRT: concurrent chemoradiotherapy;

† : ACT: adjuvant chemotherapy;

LN: lymph nodes;

†† : American Joint Committee on Cancer;

§ : R0: no residual tumor after resection;

∥ :R1: microscopic residual tumor after resection;

¶ :D2: removal of all invaded N2 lymph nodes:

# :D1:removal of all invaded N1 lymph nodes;

CCT: concurrent chemotherapy.

### 2 Treatment details

**Figure 1:**
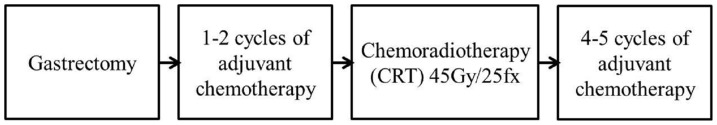
The flow chart of the recommended treatment strategy.

The recommended treatment protocol in our institute is radical resection followed by 1–2 cycles of ACT, postoperative CRT, and 4–5 cycles of ACT, as shown in Figure 1.

#### 2.1 Surgery

Surgery was performed with either total or subtotal gastrectomy. This procedure entailed the resection of all perigastric lymph nodes and some celiac, splenic or splenic-hilar, hepaticartery, and cardial lymph nodes, depending on the location of the tumor in the stomach. The regional lymph node areas were defined according to the definitions of the Japanese Research Society for Gastric Cancer [14], [15]. Pathologic staging was based on the 2002 American Joint Committee on Cancer (AJCC) TNM staging system [16].

#### 2.2 Radiotherapy

All but four patients completed adjuvant radiotherapy (RT) with or without sufficient postoperative ACT. The prescribed irradiation dose was 45–50.4 Gy with 25–28 fractions, 1.8 Gy per fraction, five days per week. The target volume included the tumor bed, anastomotic stoma, gastric remnant (T3, T4), and regional draining lymph nodes. The tumor bed was defined by preoperative and postoperative CT imaging, barium radiography, and, in some instances, surgical clips [11]. Perigastric, celiac, local paraaortic, splenic, hepatoduodenal or hepatic-portal, and pancreaticoduodenal lymph nodes were included in the radiation target volume, and the paracardial and paraesophageal lymph nodes were also included if the patient had tumors in the gastroesophageal junction. Exclusion of the splenic nodes was allowed in patients with antral lesions to protect the left kidney [17]. Some patients used an active breath coordinator (ABC, n = 15) to reduce respiration-related uncertainty during simulations and treatments. All patients were fixed with vacuum pad during simulation and treatment (CIVIC Medical solution, Iowa). Treatments were delivered using 3-dimensional conformal radiation therapy (3D-CRT, n = 93) or intensity modulated radiation therapy (IMRT, n = 4) with a 6-MV photon. Simulating CT data sets were acquired in the CT simulator (Philips Medical Madison, WI) with a 5-mm slice thickness at least 3 hours after meals. All treatment plans were optimized with a commercial treatment planning system (TPS, Philips Radiation Oncology Systems, Pinnacle version 8.0 m, Milpitas, CA). All treatments were delivered with an Elekta Synergy Slinear accelerator equipped with an electronic portal imaging device (EPID) and a kilovoltage cone-beam CT system (Elekta Synergy S, Elekta Oncology Systems, Crawley, UK). The tolerances of normal tissues were defined as follows: 1) less than 30% of the liver receiving 30 Gy, mean dose to liver less than 23 Gy; 2) less than 50% of the kidneys receiving 15 Gy, mean doses to both kidneys less than 16 Gy; and 3) less than 40% of the heart receiving 40 Gy.

#### 2.3 Chemotherapy

According to the guidelines of our institute, all patients were recommended to receive radiotherapy with concurrent chemotherapy (5-fluorouracil or capecitabine) and four to six cycles of epirubicin-based triplet adjuvant chemotherapy both before (1–2 cycle) and after (4–5 cycles) chemoradiation.

#### 2.4 Concurrent chemotherapy

Fluorouracil (225 mg/m^2^/day) was administered with continuous intravenous or oral capecitabine (625 mg/m^2^, bid) concurrently with radiation.

#### 2.5 Adjuvant chemotherapy

All patients were recommended to receive four to six additional cycles of ACT (ECF) consisting of epirubicin (50 mg/m^2^ on Day 1), cisplatin (30 mg/m^2^ on Days 1–3), and 5-FU (425 mg/m^2^/day on Days 1–5) or a modified ECF regimen consisting of either epirubicin (50 mg/m^2^ on Day 1), oxaliplatin (130 mg/m^2^ on Day 1) and 5-FU (425 mg/m^2^/day on Days 1–5) (EOF) or epirubicin (50 mg/m^2^ on Day 1), oxaliplatin (130 mg/m^2^ on Day 1), and capecitabine (625–825 mg/m^2^/day, twice a day, orally on Days 1–14) (EOX). Patients were classified into two groups according to the number of ACT cycles. The 61% (59/97) of patients who received 4–6 cycles of ACT were recorded as group 1, and the remaining 39% (38/97) were recorded as group 2.

### 3 Follow-up

All patients were assessed every 3 months for the first 3 years after completion of treatment and every 6 months for 3 additional years thereafter. Follow-up examinations, including history and physical examination, complete blood count, serum chemistry, ultrasonography of the liver, and a chest X-ray, were routinely performed by either the attending radiation oncologist or the surgeon at each follow-up session. CT scans of the chest and abdomen and upper digestive track endoscopy were performed routinely every 6 months. CT scans of the chest, CT scans or ultrasonography of the abdomen and pelvis, endoscopy of the upper digestive tract, and/or positron emission tomography (PET) were immediately performed if any symptom of disease occurred or elevated tumor marker levels were detected.

### 4 Adverse effects evaluation

Acute and late toxicities were graded according to CTCAE 3.0 and the Radiation Therapy Oncology Group/European Organization for Research and Treatment of Cancer (RTOG/EORTC) criteria [18], [19]. Late toxicities were defined as symptoms first occurring or lasting >90 days after the beginning of RT. Due to the difficulty in differentiating the underlying toxicities from surgery, CRT or ACT, these toxicities were reported together.

### 5 Statistical analysis

The sites of relapse were classified as a local recurrence if a tumor was detected in the radiation CTV volume (surgical anastomosis, residual stomach, or gastric bed and some regional lymph nodes), as regional if a tumor was detected in the peritoneal cavity (including other intra-abdominal lymph nodes and the peritoneum), and as distant if the metastases were outside the peritoneal cavity and the liver. All patients were included in the analysis of toxic effects survival rate.

The intervals until local recurrence, regional carcinomatosis, and distant metastasis were measured from the completion of surgery to the documented treatment failure. The overall survival duration was calculated from the surgery completion until death or the date of the last follow-up visit for patients still living. The Kaplan-Meier method [20] was used to estimate the DFS, OS, and local control rates. The association between each of the potential prognostic factors and the estimated local control rate, DFS, or OS was tested with the log–rank test [21]. Multivariate analysis was performed using the Cox regression model [22]. All statistical analyses were performed using SPSS 13.0 software.

## Results

Within the patient cohort, 51 had previously undergone standard D2 dissection (removal of all invaded N2 [15] lymph nodes), and the rest had undergone D1 dissections (removal of all invaded N1 [15] lymph nodes). The median number of removed lymph nodes was 16. Positive lymph nodes ratio (positive lymph nodes/total lymph nodes removed) was well balanced between each patients subgroup, and the median positive lymph nodes ratio in group1 and group2 were 0.36 (0–1) and 0.33 (0–0.96) respectively. The median follow-up time for all patients was 44 months (range 10–99 months). Of the 97 patients, 93 (95.9%) completed the entire radiotherapy without interruption, while 4 patients did not complete the radiotherapy due to acute toxicity (vomiting). Seventy-seven patients completed concurrent chemotherapy. Among the 20 patients who did not complete concurrent chemotherapy, therapy was discontinued in 5 because of acute toxicity, and the others refused to undergo concurrent chemotherapy because of the fear of adverse effects. The detailed grouping was as follows: RT alone, 2; RT and CT, 18; concurrent RTCT and CT, 41; and CRT, 36.

### 1 Treatment Outcomes

**Figure 2:**
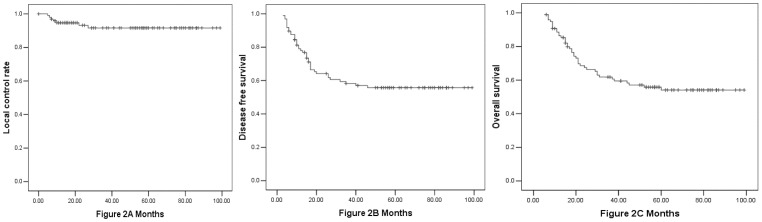
The local control rate, disease-free survival (DFS), and overall survival (OS) of the cohort (Fig. 2A: local control rate, Fig. 2B: DFS, Fig. 2C: OS).

**Figure 3:**
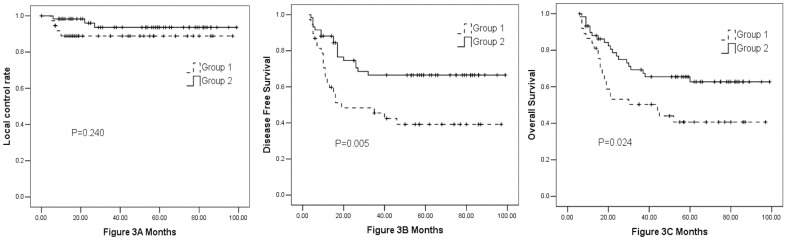
The local control rate, disease-free survival (DFS), and overall survival (OS) of patients in group 1 (0–3 cycles chemotherapy) and group 2(4–6 cycles chemotherapy) (Fig. 3A: local control rate, Fig. 3B: DFS, Fig. 3C: OS).

The 3-year local control rate, DFS, and OS were 90%, 58%, and 62%, respectively (Figure 2). Forty-one patients (42%) had disease relapse (19 in group 1 and 22 in group 2), and 40 patients (41%) died (18 in group 1 and 22 in group 2). Local recurrence developed in 5 patients (4%) in the CRT (CRT+ACT) group. A total of 14 patients had distant metastasis, 9 of whom had <4 and 5 of whom had ≥4 cycles of ACT. In addition, 15 and 12 patients developed regional peritoneum carcinomatosis in the CRT and CRT+ACT groups, respectively. The 3-year overall survival and disease-free survival were 69.5% and 66.5% for group 1 and 50% and 45.5% for group 2, respectively (p = 0.024 and p = 0.005). The local control rates were 94% and 89% for groups 1 and 2, respectively (p = 0.24) (Figure 3).

### 2 Prognostic Factors

The effects of age, gender, pathological T and N categories, positive lymph node ratio, completeness of surgery, lymphovascular invasion, and ACT on local control, peritoneal carcinomatosis, distant metastasis, DFS, and OS were evaluated by univariate and multivariate analyses (Tables 2 and 3). Patients' weight loss was not included in the balanced factors, because this study was retrospective and weight loss could be influenced by many factors, such as nutritional support and recovery condition from surgery. For patient under nutrition risk, we recommended nutritional support. Multivariate analysis revealed that number of cycles of ACT (4–6 vs 0–3) and lymphovascular invasion were independent prognostic factors for DFS (p = 0.010 and p = 0.003, respectively), while peritoneal metastasis was an independent prognostic factor for OS (p<0.01).

**Table 2:** Univariate analyses of overall survival, local control, and disease-free survival.

Variable	P				
	Overall survival	Locoregional control	Peritoneal metastasis	Remote metastasis	Disease-free survival
Age (<50 vs. ≥50 y)	0.989	0.561	0.469	0.410	0.707
Gender (male vs. female)	0.182	0.362	0.370	0.609	0.459
Location (cardia vs. body vs. antrum)	0.477	0.086	0.406	0.538	0.344
ACT (<4 vs. ≥4 c)	0.024	0.240	0.023	0.305	0.005
pT (T1–T2 vs. T3–T4)	0.098	0.181	0.564	0.218	0.264
pN (N0 vs. N1 vs. N2 vs. N3)	0.000	0.037	0.255	0.074	0.000
pN (N0–N1 vs. N2–N3)	0.011	0.007	0.132	0.082	0.013
Resection (R0 vs. R1)	0.740	0.514	0.287	0.323	0.736
lymphovascular(+vs.−)	0.001	0.170	0.004	0.047	0.000

**Table 3:** Multivariate analysis of local control, disease-free survival, and overall survival.

Variable	P				
	Overall survival	Locoregional control	Peritoneal metastasis	Remote metastasis	Disease-free survival
ACT (<4 vs. ≥4 c)	0.473	0.903	0.040	0.371	0.010
pN (N0 vs. N1 vs. N2 vs. N3)	0.489	0.717	0.411	0.177	0.098
pN (N0–N1 vs. .N2–N3)	0.587	0.152	0.880	0.423	0.681
lymphovascular (+ vs.−)	0.525	0.487	0.019	0.163	0.003

### 3 Adverse Effects

The toxicity that was observed among the 97 patients is summarized in Table 4. The major toxic effects were hematological and gastrointestinal. The most common hematologic toxic effect was leukopenia, which developed in 30 patients (31%), while severe thrombocytopenia was uncommon (2%). One patient (1%) died due to adjuvant chemotherapy-induced liver failure as well as febrile neutropenia, and another patient experienced anastomotic stoma edema 3 months after completion of the chemoradiation. Radiation-induced Grade 3 or greater acute digestive toxic effects, such as nausea/vomiting, dyspepsia, and diarrhea, were observed in 23 patients (23%). Gastrectomy-induced complications, such as anastomosis fistula, pancreatic necrosis, or wound dehiscence, were not observed.

**Table 4:** Cumulative incidence of Grade 3+ acute complications to NCI CTC 3.0 (during ACT and CRT).

Toxicity: Grade 3/4	n (%)
**Gastrointestinal**	**23 (23)**
Nausea	10 (10)
Vomiting	6 (6)
Diarrhea	2 (2)
Dysphagia	1 (1)
Anorexia	4 (4)
**Hematologic**	**36 (36)**
Neutropenia	30 (31)
Thrombocytopenia	2 (2)
Anemia	2 (2)
Febrile neutropenia	2 (2)
**Other**	**5 (5)**
Fatigue	5 (5)

## Discussion

The treatment outcomes, as indicated by DFS and OS, remain dismal for patients with advanced gastric adenocarcinoma. Complete tumor resection with sufficient lymph node dissection is the most important factor for disease control and long-term survival of gastric cancer patients.

Clearance of locoregional disease with surgery alone (even D2) is not sufficient for most patients with locally advanced gastric adenocarcinoma, and adjuvant treatment is imperative for further increasing tumor control and improving treatment outcomes. Various strategies, including neoadjuvant and/or ACT, radiation therapy, and their combination, have been studied in an attempt to improve treatment outcomes. Locoregional control has improved as radiotherapy has become one of the treatment methods for gastric cancer [23], and even DFS and OS were increased in an INT 0116 study and its update report with concurrent postoperative CRT [11], [24]. However, only 10% of patients in that study underwent D2 resection, and it was unclear whether the postoperative CRT simply compensated for the insufficient resection. Two recent studies in Korea reported that adjuvant CRT after D2 gastrectomy significantly improved treatment outcomes in patients with non-metastatic gastric adenocarcinoma [25], [26]. Therefore, postoperative concurrent CRT is an effective treatment for locoregional advanced gastric cancer.

However, in the INT 0116 study, postoperative CRT did not significantly decrease regional relapse or distant relapses. In fact, 72% of those in the surgery-only group and 65% of those in the chemoradiotherapy group had regional relapse, and 18% of those in the surgery-only group and 33% of those in the chemoradiotherapy group had distant relapse. The efficacy of neoadjuvant plus ACT with an epirubicin-based regimen was reported in the MAGIC trial [12]. The overall survival rates were 36.3% and 23.0% for patients treated with or without postoperative chemotherapy, respectively. These studies and the frequency of recurrence after resection of gastric cancer encouraged us to study the combination of CRT and adjuvant epirubicin-based chemotherapy in patients with adenocarcinoma of the stomach or gastroesophageal junction.

In the present study, patients with locally advanced gastric cancer (defined as Stage T3, T4, or N+) were treated with gastrectomy followed by CRT and ACT. These regimens substantially improved treatment outcome, including tumor control and overall survival rates. The 3-year peritoneum metastasis control and overall survival rates were 52% vs. 50% (p = 0.023) and 77% vs. 69.5% (p = 0.024) for patients with less ACT vs. more ACT, respectively. The 3-year local control rates for the CRT+ACT and CRT only groups were 94% and 89% (p = 0.240), respectively. Thus, our results are comparable to those of previous studies.

The recently reported multicenter study of the Trans-Tasman Radiation Oncology Group (TROG) [27], which included an adjuvant therapy regimen consisting of one cycle of ECF, followed by radiotherapy with concurrent infusion of 5-FU and then two additional cycles of ECF, indicated that the adjuvant regimen with ECF before and after three-dimensional conformal chemoradiation is feasible and can be safely delivered in a cooperative group setting. We found that an additional 4–6 cycles of ACT in adjuvant chemo/radiotherapy was a significant prognostic factor for peritoneal and remote control and overall survival and did not significantly increase the incidence of long-term complications. To the best of our knowledge, our report is the only analysis of this type of treatment strategy. Our results revealed lower toxicity rates, similar treatment compliance, and higher overall survival compared to those in the TROG study. The chemoradiation regimen used in our study was generally well tolerated, with 61% of patients (compared to 67% in the TROG study) completing all courses of treatment (CRT+ 4–6 cycles of ACT) and only 9% (6% in the TROG) of patients unable to complete concurrent CRT because of treatment-related toxicity. The hematological and gastrointestinal (GI) toxicity rates were also similar to those in the TROG trial. Nevertheless, the 3-year overall survival and disease-free survival were higher than those in the TROG study (61% and 70% vs. 58.6% and 61.6%, respectively), which may be due to more thorough surgical treatment and more cycles of chemotherapy. In our study, 53 patients (52%) had previously undergone formal D2 dissection, and D1 dissection (removal of all invaded N1 lymph nodes) had been performed in the rest (48%), while in the TROG study, 20% underwent less than a D1 lymph node dissection. In addition, our study included more cycles of ACT than the TROG study.

The phase III trial of ARTIST [28] compared postoperative treatment with capecitabine plus cisplatin (XP) versus XP plus radiotherapy with capecitabine (XP/XRT/XP); in this trial, the addition of XRT to XP chemotherapy did not significantly reduce recurrence after curative resection and D2 lymph node dissection in gastric cancer. In a subgroup analysis of patients with positive pathologic lymph nodes, there was a statistically significant increase in DFS in the XP/XRT/XP arm (estimated 3-year DFS rate of 77.5%) as compared to the XP-alone arm (3-year DFS, 72.3%; P = .0365). In this study, 60% of patients were staged Ib or II, and relatively early stage patients may have a better prognosis and may not benefit as much from adjuvant treatment. This may also explain the increased DFS in that study compared to that in the present study. A French retrospective study [29] concluded that postoperative cisplatin-based chemotherapy followed by conformal radiotherapy with concurrent 5-FU was feasible. The overall and disease-free survival rates were comparable to those previously reported in the literature, with good local and regional disease control. Despite greater use of postoperative chemotherapy with cisplatin, distant and peritoneal recurrences remain the most frequently observed relapses.

The French FNCLCC (Federation Nationale des Centres de Lutte Contre le Cancer) ACCORD07-FFCD 9703 randomized trial [30] reported that preoperative 5-FU and cisplatin chemotherapy in patients with resectable adenocarcinoma of the stomach and lower esophagus significantly improved 5-year overall and disease-free survival compared with surgery alone: 38% vs. 24% and 34% vs. 21%, respectively. The CLASSIC trial [31] demonstrated that adjuvant chemotherapy with capecitabine plus oxaliplatin (8 cycles) after D2 gastrectomy significantly improved 3-year disease-free survival to 74% from 59.0% with surgery alone.

The ongoing US Intergroup trial (CALGB 80101) is a randomized, Phase III trial with an ECF-based regimen that is similar to that used in the TROG study. Patients in the Intergroup trial also receive one cycle of ECF before chemoradiation and two cycles after chemoradiation. However, due to the high rate of acute toxicity (as reported in the TROG study), the drug doses are reduced in the postradiotherapy cycles. We used a chemotherapy regimen with lower toxicity (EOF or EOX) for most patients, enabling these regimens to be better tolerated.

The Dutch/Swedish trial (Randomized Phase III Trial of Adjuvant Chemotherapy or Chemoradiotherapy in Resectable Gastric Cancer (CRITICS) study; ClinicalTrials.gov ID NCT00407186) is comparing three postoperative courses of epirubicin, cisplatin, and capecitabine chemotherapy vs. chemoradiotherapy with capecitabine and cisplatin in patients with gastric cancer treated with three preoperative courses of epirubicin, cisplatin, and capecitabine chemotherapy followed by surgery with D2 lymphadenectomy without splenectomy and pancreatectomy. A phase III trial (ARTIST-II) to compare chemotherapy versus chemotherapy with RT in patients with D2 lymph node dissection and pathologic lymph node–positive disease is planned to confirm the benefits of adjuvant CRT.

The TOPGEAR trial is investigating whether the addition of chemoradiotherapy to chemotherapy is superior to chemotherapy alone in the neoadjuvant setting by improving the initial pathological complete response (pCR) rates as well as subsequent overall survival in patients undergoing adequate surgery (D1 dissection) for resectable gastric cancer.

Several important issues remain. First, because our study was a retrospective study, the number of patients required for adequate statistical power was not determined. More broadly, as the role of neoadjuvant plus ACT in the management of advanced gastric cancer becomes clear, whether the addition of preoperative chemotherapy can further improve the treatment outcome needs to be determined. In addition, it is unclear if preoperative chemoradiotherapy plus postoperative chemotherapy is superior to our method with respect to treatment outcome and tolerance. In our study, the main recurrence observed was peritoneum metastasis, which indicated that further treatment was needed to reduce regional recurrence. Third, if an epirubicin-based chemotherapy regimen in the adjuvant chemoradiotherapy treatment is most effective, insight from the ARTIST trial [28] on the choice of postoperative adjuvant chemotherapy regimen may help to lower the toxicity rate. Finally, multivariate analysis revealed that were no significant differences in regional or remote control or overall survival rates in terms of the various prognostic factors; however, the disease-free survival showed significant differences in regional or remote control and overall survival rates in terms of the prognostic factors, as assessed by the log-rank test.

Future studies should aim to resolve these questions. The development of an optimal adjuvant chemoradiotherapy strategy for locally advanced adenocarcinoma of the stomach would be a significant achievement in the field, and collaborative efforts among cancer treatment institutions are the best strategy for reaching this goal.

## Conclusions

Gastrectomy with lymph node dissection and CRT followed by ACT is feasible and tolerable for the treatment of locally advanced gastric cancer. The addition of sufficient ACT to the postoperative CRT significantly improved 3-year abdominal carcinomatosis control and the 3-year overall survival rate. Compared to the 5-FU-based CRT strategy, this novel CRT regimen with an increased ACT regimen used in an adjuvant setting should be further optimized and studied in prospective trials to determine whether it can further improve the final treatment outcome.
